# Thoracolumbar fascia deformation during deadlifting and trunk extension in individuals with and without back pain

**DOI:** 10.3389/fmed.2023.1177146

**Published:** 2023-06-05

**Authors:** Andreas Brandl, Jan Wilke, Christoph Egner, Rüdiger Reer, Tobias Schmidt, Robert Schleip

**Affiliations:** ^1^Department of Sports Medicine, Institute for Human Movement Science, Faculty for Psychology and Human Movement Science, University of Hamburg, Hamburg, Germany; ^2^Department for Medical Professions, Diploma Hochschule, Bad Sooden-Allendorf, Germany; ^3^Osteopathic Research Institute, Osteopathie Schule Deutschland, Hamburg, Germany; ^4^Department of Movement Sciences, University of Klagenfurt, Klagenfurt, Austria; ^5^Institute of Interdisciplinary Exercise Science and Sports Medicine, Medical School Hamburg (MSH), Hamburg, Germany; ^6^Department of Sport and Health Sciences, Conservative and Rehabilitative Orthopedics, Technical University of Munich, Munich, Germany

**Keywords:** thoracolumbar fascia deformation, track and field athletes, acute low back pain, deadlift velocity, trunk extension, correlation

## Abstract

**Background:**

Alterations in posture, lumbopelvic kinematics, and movement patterns are commonly seen in patients with low back pain. Therefore, strengthening the posterior muscle chain has been shown to result in significant improvement in pain and disability status. Recent studies suggest that thoracolumbar fascia (TLF) has a major impact on the maintenance of spinal stability and paraspinal muscle activity, and thus is likely to have an equal impact on deadlift performance.

**Objective:**

Aim of the study was to evaluate the role of thoracolumbar fascia deformation (TFLD) during spinal movement in track and field athletes (TF) as well as individuals with and without acute low back pain (aLBP).

**Methods:**

A case–control study was performed with *n* = 16 aLBP patients (cases) and two control groups: untrained healthy individuals (UH, *n* = 16) and TF (*n* = 16). Participants performed a trunk extension task (TET) and a deadlift, being assessed for erector spinae muscle thickness (EST) and TLFD using high-resolution ultrasound imaging. Mean deadlift velocity (VEL) and deviation of barbell path (DEV) were measured by means of a three-axis gyroscope. Group differences for TLFD during the TET were examined using ANOVA. Partial Spearman rank correlations were calculated between TLFD and VEL adjusting for baseline covariates, EST, and DEV. TLFD during deadlifting was compared between groups using ANCOVA adjusting for EST, DEV, and VEL.

**Results:**

TLFD during the TET differed significantly between groups. TF had the largest TLFD (−37.6%), followed by UH (−26.4%), while aLBP patients had almost no TLFD (−2.7%). There was a strong negative correlation between TLFD and deadlift VEL in all groups (r = −0.65 to −0.89) which was highest for TF (*r* = −0.89). TLFD during deadlift, corrected for VEL, also differed significantly between groups. TF exhibited the smallest TLFD (−11.9%), followed by aLBP patients (−21.4%), and UH (−31.9%).

**Conclusion:**

TFLD maybe a suitable parameter to distinguish LBP patients and healthy individuals during lifting tasks. The cause-effect triangle between spinal movement, TFLD and movement velocity needs to be further clarified.

**Clinical trial registration:**

https://drks.de/register/de/trial/DRKS00027074/, German Clinical Trials Register DRKS00027074.

## 1. Introduction

The deadlift is a widely used strength and conditioning exercise of many athletes, i.e., in track and field (1–3). Electromyography studies have shown that a variety of muscles in both, the lower and upper body are involved. Among these, the erector spinae (ES) and semitendinosus muscles exhibit the highest activations (4). Coaches rely on the deadlift not only because of the contribution of many large muscle groups, but also because of their simultaneous activation (5). The neuromotor control of the entire kinematic chain and the management of significant musculoskeletal loads require a great deal of coordinative effort. As a consequence, the frequent use of deadlifts can lead to multiple systematic adaptations (5).

Because altered posture, lumbopelvic kinematics, and movement patterns are often seen in low back pain patients, resistance training is typically recommended to improve functional status (6, 7). Strengthening the posterior chain, in conjunction with general exercise, has been demonstrated to significantly improve pain and disability status (7, 8). In this regard, exercises that recruit multiple muscles of the posterior chain, such as the deadlift, seem to be most effective (8).

Panjabi (9) presented a model of the spinal stabilization system, which consists of three subsystems. Vertebrae with their disks and their ligaments, muscles and tendons attached to the spine, and the neural system. The thoracolumbar fascia (TLF) and other components have been considered only as passive surrounding tissues, if at all (10, 11). However, recent studies suggest that the TLF may have a much greater influence on the maintenance of spinal stability and likewise paraspinal muscle activity. Using magnetic resonance imaging-based finite element analysis, Bojairami et al. (12) demonstrated that the TLF alone contributes 75% to spinal static stability.

Some studies suggest that the ability of the TLF to deform (in terms of stretching and relaxing) and slide on the epimysium of the erector spinae is a critical feature that distinguishes healthy individuals from low back pain patients (13–15). This group of patients has 13.8% less shear strain (13) and 28% less TLF deformability (15) than healthy controls.

The aim of this study was to investigate TLF deformation (TLFD) in athletes and non-athletes with and without acute low back pain (aLBP). Based on a previous study, we hypothesized that untrained aLBP patients (UaLBP) would have a lower TLFD than untrained healthy participants (UH) or track and field athletes [TF; Hypothesis 1; (13)]. It was further hypothesized that a lower TLFD would correlate with a lower deadlift velocity (VEL) (Hypothesis 2). However, because of the different groups, it was assumed that these examinations would also be influenced by the different muscle training status of athletes and non-athletes and probably by different movement patterns (e.g., pain avoidance in aLBP patients). Therefore, the analysis had to control for these group differences. It was thought that muscle training status, different movement patterns, or VEL would influence TLFD during deadlift in the groups and, therefore, the adjusted TLFD would differ from the unadjusted TLFD (Hypothesis 3).

## 2. Methods

We performed a case–control study with UaLBP patient cases and two control groups (athletes and pain-free adults). The study protocol was prospectively registered with the German Clinical Trials Register (DRKS00027074). The study, which adhered to the STROBE Statement, was reviewed, and approved by the ethical committee of the Diploma Hochschule, Germany (Nr.1014/2021). It was conducted in accordance with the declaration of Helsinki and all participants provided written informed consent.

### 2.1. Participants

The study was performed in a center for manual and regenerative orthopedic medicine in a medium-sized city in southern Germany. The sample size of the three groups was calculated based on data from a previous study comparing TLFD in UH and UaLBP (Cohen’s *d* = 1.2, α err = 0.05, 1–β err = 0.9; 13). Assuming a drop-out rate of 5–10%, we enrolled *n* = 16 participants per group. Sex of participants was balanced (8 men and 8 women) in each group.

As outlined, cases were UaLBP. The acquisition for the UaLBP group was carried out *via* direct contact, a notice board, and the distribution of information material at the center. One control group consisted of UH, while the other comprised TF athletes. The UH group was recruited in a local school for manual health professions while the TF group were top national level TF athletes from the southern German TF base.

All participants were aged between 18 and 60 years. Further inclusion criteria for UaLBP were: acute lumbar back pain as defined by the European guidelines for the management of acute low back pain (16); minimum score of 10 on the Oswestry Disability Questionnaire [ODQ-D; (15)]; minimum score of 3 on the visual analog scale (VAS) for pain intensity; <6 weeks pain duration. Participants were eligible for TF if they qualified at least once for the German championships in the last 2 years, were active athletes of the southern German TF base, and had a 1 repetition maximum (1RM) of no more than 130 kg. In the UH and UaLBP groups, participants had to be naive with regard to resistance training (not more than two exercise sessions per month).

Participants were excluded in case of contraindications to deadlift exercise; rheumatic diseases; intake of muscle relaxants or drugs affecting blood coagulation or drug treatment of endocrine diseases; skin changes (e.g., neurodermatitis, psoriasis, urticaria, decubitus ulcers); surgery or other scars in the lumbar region between Th12 and S1; acute trauma; neurologic or psychiatric disorders, BMI < 18.5 or >34.9. Additional exclusion criteria for TF and UH were presence or history (no doctor or therapist visit in the past 5 years) of aLBP.

### 2.2. Experimental protocol and outcomes

**Figure 1:**
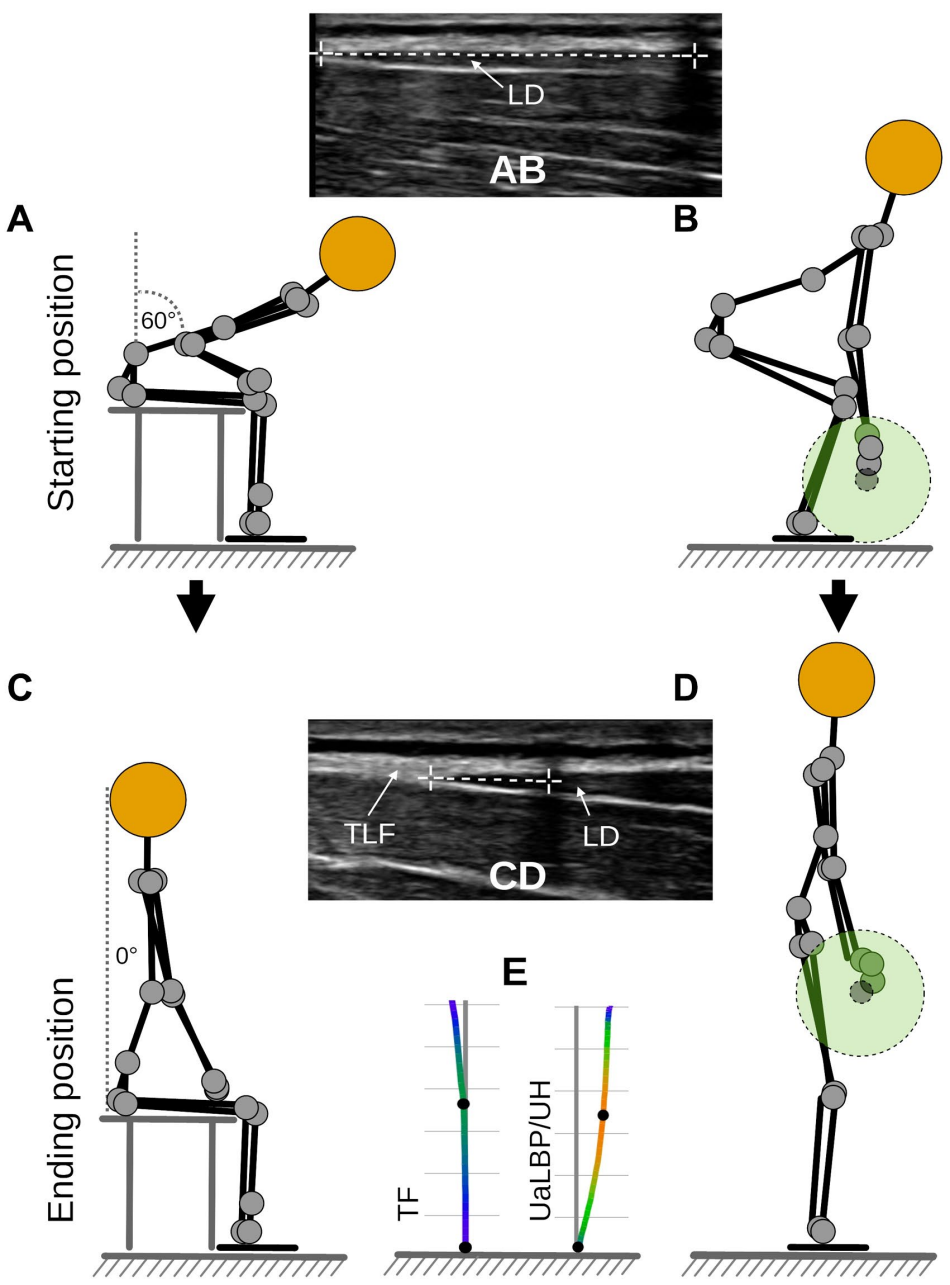
Measurement procedure. **(A)** Flexion phase trunk extension task. **(B)** Starting position for deadlift. **(A,B)** Measurement time point t_1_. **(C)** Fully extended position of trunk extension task. **(D)** Ending position for deadlift. **(C,D)** Measurement time point t_2_. **(E)** Barbell path for TF and UaLBP/UH. UaLBP, untrained low back pain patients; UH, untrained healthy subjects; TF, track and field athletes; TLF, thoracolumbar fascia; LD, latissimus dorsi muscle.

The UaLBP group completed the ODQ-D (17) and determined their current pain level using the VAS. Participants in the TF group had sufficient experience in deadlifting (checked by means of anamnesis and visual inspection by a qualified trainer) and knew their 1RM from current training logs. However, all individuals were additionally familiarized with the used conventional deadlift (using an Olympic barbell, Rogue Fitness, Columbus, US) 2 weeks, 1 week, and 1 day prior to the actual data collection. The conventional deadlift used was described in detail by Graham (1) and Farley (2). The movement starts with the feet shoulder-width apart and an alternating barbell grip. The first pull is initiated by extending the hip and knee joints simultaneously. While keeping the body weight over the center of the feet, the barbell is held as close to the shins as possible and lifted at the highest possible speed. The movement is completed by bringing the spine into a fully upright, natural position. Participants were given further information on how to perform a defined trunk extension task (TET) from a 60° flexed hip position, as described in a previous study (15). Briefly, participants seated on a treatment table first performed a 60-degree thoracolumbar flexion which was controlled using a digital goniometer. Subsequently, they extended the trunk over 8 s to the neutral position. Ultrasound measurement of TLFD was performed in the starting and ending positions as in deadlift (Figures 1A–D). In this regard, the investigator demonstrated a complete cycle of this TET (Figures 1A,C). The same person instructed the exercises for each participant.

Prior to the test session, participants performed three warm-up sets of 5 repetitions with 20 kg. Dynamic ultrasound measurements (Philips Lumify linear transducer L12-4, 12 MHz; Philips Ultrasound Inc., Bothell, WA) of the TLFD between the latissimus dorsi muscle junction and an artificial reference using a reflective tape were performed in the starting and ending positions [Figures 1A–D,; (16)]. This approach is described in detail by Brandl et al. (15). TLFD was defined as the difference between both measured positions. This approach achieved excellent validity compared to marker-based, three-dimensional methods [ICC = 0.97; (16)]. The mean VEL and deviation of barbell path (DEV) were determined using a three-axis gyroscope (Vmaxpro; Blaumann & Meyer, Sports Technology UG, Magdeburg, Germany) magnetically attached to the center of the barbell (18). The Vmaxpro sensor was validated and showed a velocity prediction accuracy of 99% for the deadlift (18). The minimum detectable difference at loads above 20% 1RM is 0.1 m/s (19). Hence, we used a load of 40 kg (30% of the maximum included 1RM) and set a maximum 1RM of 130 kg (100% of the maximum included 1RM) as the inclusion criterion for TF. The correlation of actual 1RM and 1RM predicted by VEL using linear regression is *r* = 0.97, *p* < 0.05 (20). Participants repeated three 40 kg deadlifts under motivating cheering of the instructor, and the one with the highest achieved VEL was used for further analysis. DEV was calculated as the sum of the highest deviation in meter (anterior, posterior, right, and left deviation) from a vertical axis of 90° to the floor, from the starting to the ending position of the barbell during this deadlift (Figure 1E). Compared to a 3D motion capture system, which is considered the gold standard, the Vmaxpro sensor correlated almost perfectly (*r* = 0.99) in movement detection and velocity calculation based on it (21).

The order of the deadlift or TET and the side of ultrasound measurement were randomly assigned to the participants using the Research Randomizer, version 4.0 (22). After completion of both tasks, ES thickness (EST) was measured as distance between superficial and deep aponeurosis between L3 and L4 according to the protocol of Cuesta-Vargas et al. (23) in an upright sitting position on the randomly determined side.

### 2.3. Statistical analysis

Mean, standard deviation, and 95% confidence interval (95% CI) were determined for all parameters.

TLFD during TET between groups was examined using a one-way ANCOVA (Hypothesis 1). Although age at baseline was not significantly different between groups, there was a trend toward a mismatch (*p* = 0.05). Therefore, the statistic was controlled for age as a covariate. Significant results were analyzed *post hoc* using Tukey’s HSD test. The outcome variables were normally distributed as assessed by the Shapiro–Wilk test (*p* > 0.05). The homogeneity of the error variances between the groups was fulfilled for all these variables according to Levene’s test (*p* > 0.05).

Spearman correlation coefficients were calculated for the non-normally distributed data assessed with the Shapiro–Wilk test (*p* < 0.05) to detect possible monotonic relationships between TLFD and VEL during deadlift (Hypothesis 2). Both full and partial correlations, adjusting for baseline covariates (sex, age, BMI), EST and DEV were calculated. Resulting values were interpreted according to Cohen [44] as ‘weak’ (>0.09, <0.30), ‘medium’ (>0.29, <0.50), and ‘strong’ (≥0.50).

A one-way ANCOVA was conducted to compare TLFD during deadlifting between groups, controlling for the influence of the covariates VEL, EST, DEV, and age (Hypothesis 3). The post-hoc test for a significant result was performed using Tukey’s HSD test. Estimated marginal means, standard errors, and their 95% CIs were calculated. The data were normally distributed as assessed by the Shapiro–Wilk test (*p* > 0.05). The criterion of homogeneity of the error variances between the groups was fulfilled according to Levene’s test (*p* > 0.05). Assumption of homogeneity of regression slopes was not violated with regard to the dependent variable (group), as the interaction terms were not statistically significant (*p* > 0.05).

All analyses were performed using Jamovi 2.3 (The jamovi project^1^).

## 3. Results

**Figure 2:**
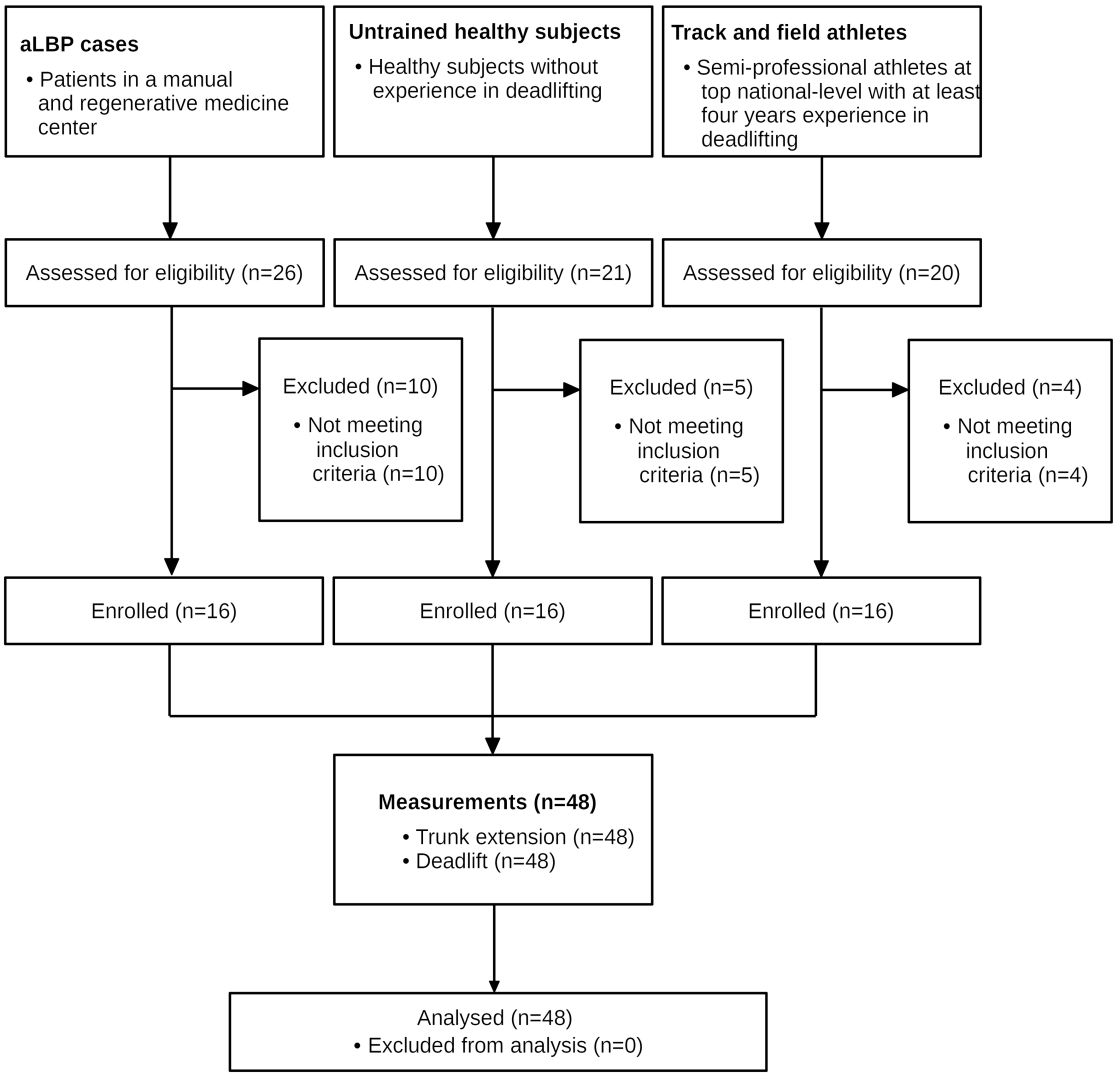
Study flow.

The anthropometric data and baseline characteristics are shown in Table 1. As per the *a priori* sample size calculation, *n* = 48 participants (16 TF, 16 UH, 16 UaLBP) took part in the study (Figure 2). No adverse events or drop-outs were recorded.

**Table 1:** Baseline characteristics.

Baseline characteristics	UaLBP (*n* = 16)		UH (*n* = 16)		TF (*n* = 16)		
	M ± SD	95% CI	M ± SD	95% CI	M ± SD	95% CI	P
Sex (men/woman)	8/8		8/8		8/8		
Age (years)	42.4 ± 10.3	36.9–47.8	41.4 ± 13.3	34.2–48.4	32.6 ± 12.0	26.2–39.0	0.05
Weight (kg)	66.4 ± 8.7	61.8–71.0	66.5 ± 8.7	61.8–71.1	65.8 ± 8.1	61.5–70.1	0.97
Height (m)	1.69 ± 0.08	1.65–1.77	1.70 ± 0.07	1.66–1.74	1.76 ± 0.07	1.69–1.76	0.40
BMI (kg/m^2^)	23.1 ± 2.3	21.9–24.4	23.1 ± 3.2	21.4–24.8	22.0 ± 1.8	21.1–22.9	0.25
Erector spinae thickness (mm)	30.1 ± 6.0	26.9–33.2	29.8 ± 3.5	27.9–31.7	40.0 ± 3.9	37.9–42.1	<0.01
Deadlift velocity (m/s)	0.62 ± 0.09	0.57–0.67	0.81 ± 0.11	0.75–0.87	1.04 ± 0.20	0.94–1.16	<0.01
Deviation of barbell path (m)	0.15 ± 0.05	0.12–0.17	0.13 ± 0.05	0.10–0.15	0.03 ± 0.02	0.02–0.04	<0.01
1 Repetition maximum (kg)					101 ± 22.2	89–113	
ODQ-D (0–100)	61.3 ± 19.1	51.2–71.5					
VAS (0–10)	6.5 ± 2.5	5.2–7.8					
Pain duration (days)	10.5 ± 8.7	5.9–15.1					

UaLBP, untrained acute low back pain patients; UH, untrained healthy participants; TF, track and field athletes; n, number; M, mean; SD, standard deviation; 95% CI, 95% confidence interval; P, *P*-Value of the one-way ANOVA; BMI, body mass index; ODQ-D, Oswestry disability questionnaire in the German version; VAS, visual analog scale.

**Figure 3:**
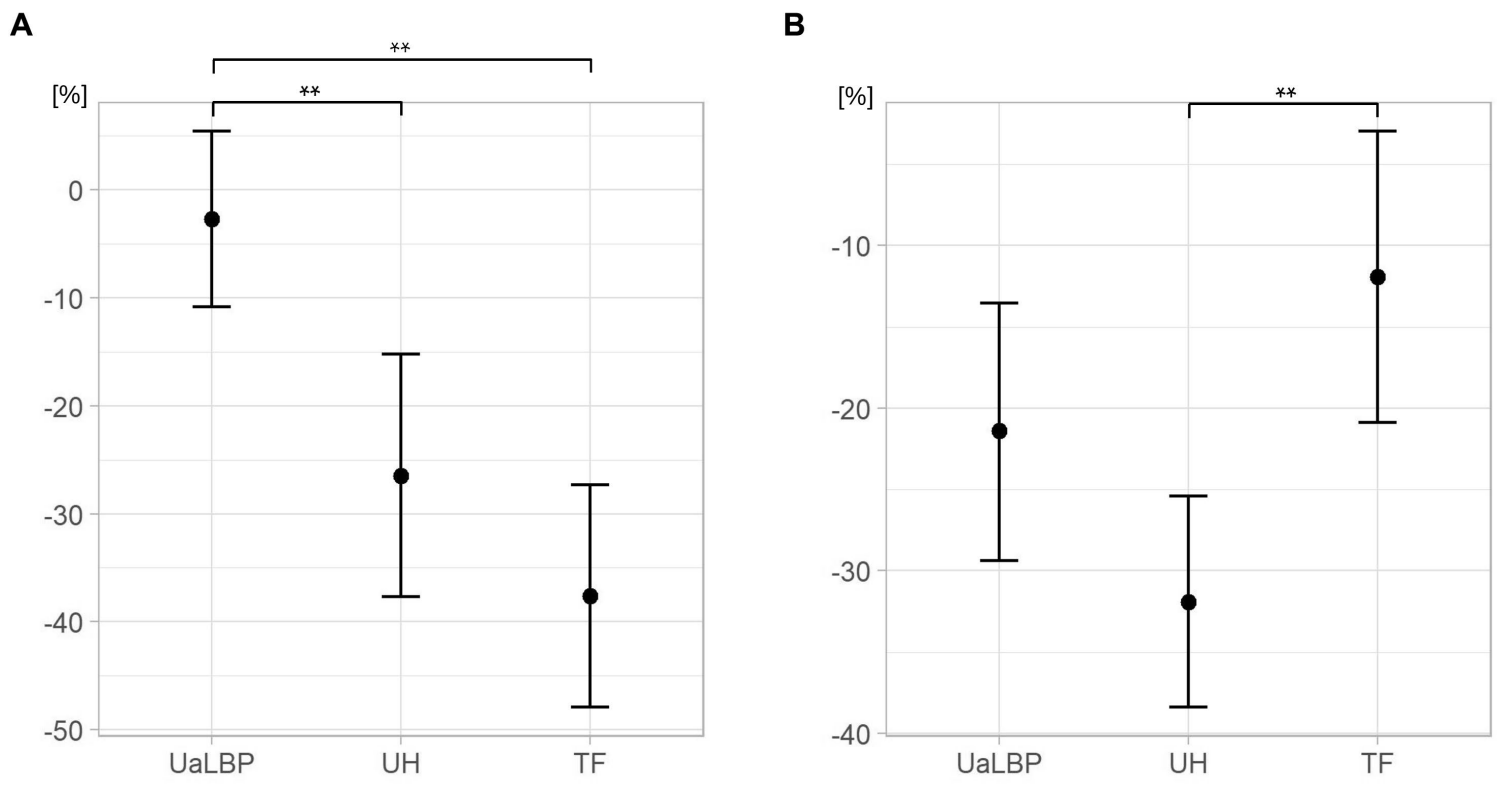
Group comparison of deformation of thoracolumbar fascia. **(A)** During a trunk extension task. Points show the mean. **(B)** During deadlift. Points show estimated marginal means corrected for deadlift velocity, barbell deviation, and erector spinae thickness. UaLBP, untrained acute low back pain patients; UH, untrained healthy participants; TF, track and field athletes. Error bars show the 95% confidence interval. Significant at the level ** < 0.01.

The one-way ANCOVA revealed significant differences regarding TLFD during TET between groups [*F*(2, 44) = 11.7; *p* < 0.01; partial η^2^ = 0.35; Hypothesis 1]. There was no univariate effect of the covariate age [*F*(1, 44) = 2.0, *p* = 0.17, η^2^ = 0.04]. Tukey’s HSD showed significant differences between UaLBP and UH (24%; *p* < 0.01) and UaLBP and TF (35%; *p* < 0.01), but not between UH and TF (11%; *p* = 0.22). Descriptive statistics are shown in Figure 3A and Table 2A.

**Table 2:** Group comparison of deformation of thoracolumbar fascia.

Variable	UaLBP (*n* = 16)		UH (*n* = 16)		TF (*n* = 16)	
	M ± SD/SE	95% CI	M ± SD/SE	95% CI	M ± SD/SE	95% CI
(A) Trunk extension task						
TLF deformation (%)	−2.7 ± 15.3^1^	−10.0 to −0.2	−26.4 ± 21.0^1^	−48.0 to −8.8	−37.6 ± 19.3^1^	−61.0 to −21.0
(B) Deadlift						
TLF deformation (%)	−3.2 ± 15.3^1^	−11.3 to 5.0	−25.3 ± 19.0^1^	−35.3 to −15.1	−37.0 ± 19.1^1^	−47.0 to −26.7
TLF deformation adjusted^3^ (%)	−21.4 ± 3.2^2^	−29.4 to −13.5	−31.9 ± 3.2^2^	−38.4 to −25.4	−11.9 ± 4.6^2^	−20.9 to −3.0

UaLBP, untrained acute low back pain patients; UH, untrained healthy participants; TF, track and field athletes; n, number; M, mean; SD, standard deviation; 95% CI, 95% confidence interval; ^1^SD, standard deviation; ^2^SE, standard error; ^3^estimated marginal means corrected for deadlift velocity, barbell deviation, and erector spinae thickness and their 95% confidence interval.

There were strong negative correlations between TFLD and VEL during deadlift for UaLBP [*r*_s(14)_ = −0.81, *p* < 0.01], UH [*r*_s(14)_ = −0.88, *p* < 0.01], and TF [*r*_s(14)_ = −0.89, *p* < 0.01]. The partial correlations were also strong for UaLBP [*r*_s (14)_ = −0.65, *p* = 0.04], UH [*r*_s (14)_ = −0.68, *p* = 0.03], and TF [*r*_s (14)_ = −0.89, *p* < 0.01] (Hypothesis 2).

The ANCOVA for group comparisons of TLFD during deadlift revealed significant differences [*F*(2, 42) = 6.78; *p* < 0.01; partial η^2^ = 0.24; Hypothesis 3]. There was a univariate effect of the covariates VEL [*F*(1, 42) = 18.2, *p* < 0.01, η^2^ = 0.30] and EST [*F*(1, 42) = 10.0, *p* < 0.01, η^2^ = 0.19] but no significant effect of DEV [*F*(1, 42) = 0.20, *p* = 0.66, η^2^ = 0.005] or age [*F*(1, 42) = 0.42, *p* = 0.52, η^2^ = 0.01]. Tukey’s HSD showed a significant difference between UH and TF (−20%; *p* < 0.01), but not between UaLBP and UH (10%; *p* = 0.09), and UaLBP and TF (−10%; *p* = 0.39). Descriptive statistics are shown in Figure 3B and Table 2B.

## 4. Discussion

The basic mechanisms of force transmission from fascial tissue to skeletal muscle have been studied and convincing evidence of this has been found (24–26). However, the relative contributions of these mechanical interactions to training outcomes or remote exercise effects under *in vivo* conditions are often neglected in sports science as noted by a consensus paper, pointing to the urgency of research in this area. (27). The present study is novel in that it examined TLFD (measured with ultrasound) and deadlift velocity (measured with a three-axis gyroscope) during spinal movement in athletes and non-athletes.

One of the main findings of this study was a high TLFD in UH (−26%) and TF (−37%) in contrast to UaLBP (−3%) during the TET (Hypothesis 1). This confirms previous research in which the TLF was 28% less deformable in UaLBP than in healthy participants (15). In conjunction with the earlier finding of reduced shear strain between the fascial layers of the TLF in low back pain, this may be indicative of adhesions between the TLF and the epimysium of the ES (13). It has been suggested that impaired neuromuscular control and recruitment patterns due to low back pain lead to this observation (13). However, the results of our study tend to imply that TLFD could also be dependent on training status and activity and it becomes apparent that the understanding of these relationships needs to be extended in future studies.

To determine the presumed correlations between TLFD, and VEL without the influence of the epidemiological variables, EST and DEV, a partial correlation was performed which corrected the related calculations (Hypothesis 2). TLFD showed a strong correlation with VEL (*p* < 0.01), which was highest level [*r*_s(14)_ = 0.88] in TF. This was surprising because the EST, together with the pennation angle of the ES fibers, predicts the torque that the muscle can deliver by 68% (23). Therefore, it was hypothesized that the correlation corrected for EST would be less than the full correlation. However, finite model analysis suggests that the ES can only exert its full force with the support of TLF [by decreasing the intramuscular pressure and thereby reducing the stress; (12)], which could explain these results and the linear relationship between TLFD and VEL. Future studies focusing on the fascial system in both competitive sports and pathological changes in the myofascial system are therefore promising.

There was a significant difference between TF and UH in TFLD during the deadlift adjusting for EST, DEV, and VEL but no differences between UaLBP and the other groups (Hypothesis 3). The ANCOVA revealed a significant effect for VEL and EST (*p* < 0.01) but not for DEV (*p* = 0.66). The estimated marginal means of TF differed the most from the unadjusted mean (−11.9% versus −37.0%), suggesting that training status and velocity during the deadlift contributed significantly to TLFD during the exercise. TF had an about 20% thicker ES, achieving a 33–62% higher speed in the deadlift than UH or UaLBP. For UaLBP, the estimated marginal means showed exactly the opposite difference from the unadjusted means of TF (−21% versus −3.2%). The EST at baseline was almost the same in UaLBP and UH (30.1 mm versus 29.8 mm), which is consistent with a previous study that found no differences in deadlift force or electromyographic excitation between aLBP patients and healthy controls (8). A lower VEL is commonly observed in individuals with aLBP and has been associated with avoidance of potentially pain-causing movements (7). The UaLBP in this study achieved only 75% of the VEL of UH and 61% of TF. This could confirm this assumption. Despite an indication of reduced mean lumbar ROM in a meta-analysis by Laird et al. (7), the ANCOVA revealed no significant influence of DEV on TLFD during deadlift. However, the studies included in the analysis by Laird defined low back pain inconsistently. Therefore, it is possible that in the acute state, this reduced ROM is less significant.

### 4.1. Limitations

Our trial has some shortcomings. First, the weight was not normalized to maximal strength in the deadlifting task. Possibly this explains the differential findings to the TET task. However, the velocity determined by the Vmaxpro sensor correlates strongly with the 1RM [*r* = 0.97; (20)]. Therefore, further studies need to clarify the direction of the effect of VEL on TFLD or vice versa. Second, TLFD was not corrected for electromyographic muscle activity, which may have slightly influenced the measurements. In a previous study, no significant Granger causality of erector spinae muscle activity on TLFD was found (15), but this has to be confirmed in further studies. Third, age at baseline showed a non-significant trend toward a younger sample in the TF group. It is well known that muscle performance, morphology (28), and connective tissue (29) exhibit age-related changes. The statistics were therefore controlled for age and did not show a univariate effect in the analysis. It is therefore considered that the sampling in this study was sufficient to exclude age-related bias.

## 5. Conclusion

The TLFD during a TET is lower in UaLBP compared to UH and TF who achieved the highest TLFD. TLFD and velocity performance during deadlift are strongly correlated. The training status, respectively, the force of ES and VEL that a participant can exert on the dumbbell during the deadlift, in addition to the group membership, determines the TLFD during the exercise. The results suggest that TLFD, in addition to training status, may be an important factor in weightlifting or spinal extension tasks.

## Data availability statement

The raw data supporting the conclusions of this article will be made available by the authors, without undue reservation.

## Ethics statement

The studies involving human participants were reviewed and approved by the Diploma University of Applied Science. The patients/participants provided their written informed consent to participate in this study.

## Author contributions

AB, RR, TS, and RS designed the study. AB wrote the first draft of the manuscript. AB, JW, RR, TS, and RS co-wrote and reviewed the manuscript. AB and JW performed the statistical analysis. All authors read and approved the final manuscript.

## Conflict of interest

The authors declare that the research was conducted in the absence of any commercial or financial relationships that could be construed as a potential conflict of interest.

## Publisher’s note

All claims expressed in this article are solely those of the authors and do not necessarily represent those of their affiliated organizations, or those of the publisher, the editors and the reviewers. Any product that may be evaluated in this article, or claim that may be made by its manufacturer, is not guaranteed or endorsed by the publisher.

## Footnotes

^1^ https://www.jamovi.org
